# Histone Modifications and Non-Coding RNAs: Mutual Epigenetic Regulation and Role in Pathogenesis

**DOI:** 10.3390/ijms23105801

**Published:** 2022-05-22

**Authors:** Irina V. Bure, Marina V. Nemtsova, Ekaterina B. Kuznetsova

**Affiliations:** 1 Laboratory of Medical Genetics, I. M. Sechenov First Moscow State Medical University (Sechenov University), 119991 Moscow, Russia; nemtsova_m_v@mail.ru (M.V.N.); kuznetsova.k@bk.ru (E.B.K.); 2 Laboratory of Epigenetics, Research Centre for Medical Genetics, 115522 Moscow, Russia

**Keywords:** histone modifications, non-coding RNAs, epigenetics, biomarker

## Abstract

In the last few years, more and more scientists have suggested and confirmed that epigenetic regulators are tightly connected and form a comprehensive network of regulatory pathways and feedback loops. This is particularly interesting for a better understanding of processes that occur in the development and progression of various diseases. Appearing on the preclinical stages of diseases, epigenetic aberrations may be prominent biomarkers. Being dynamic and reversible, epigenetic modifications could become targets for a novel option for therapy. Therefore, in this review, we are focusing on histone modifications and ncRNAs, their mutual regulation, role in cellular processes and potential clinical application.

## 1. Introduction

In recent decades, it became apparent that epigenetic changes play an important role both in normal organism functioning and in disease progression. They are described as mechanisms that can lead to inherited changes in phenotype or gene expression but do not involve the DNA sequence. Among the epigenetic regulators are DNA methylation/demethylation, chromatin remodeling, histone modifications and non-coding RNAs (ncRNAs) [1]. Moreover, it is increasingly clear that they do not play all alone but regulate gene expression simultaneously, combining in a wide regulatory network [2,3,4].

DNA methylation is a process of selective addition of a methyl group to cytosine residues within CpG dinucleotides. It is established and maintained by three types of DNA methyltransferases (DNMTs), namely DNMT1, DNMT3A and DNMT3B [5]. DNA methylation is generally associated with gene silencing, and aberrations in methylation patterns lead to different types of cancer and other diseases [6].

Another mechanism of epigenetic regulation of gene expression is a “histone code”, which involves post-translational modifications of amino-acid sequences of the histones in its flexible parts that protrude from the nucleosome core (histone tails) through acetylation, methylation and other reversible chemical modulations [7]. These covalent post-translation modifications control chromatin state and thus regulate gene transcription. Moreover, each modification demonstrates specific functions [8]. There are the active histone marks that are associated with high gene expression and repressive histone marks, which are correlated with heterochromatin and gene repression [9,10]. According to the “histone code” hypothesis, numerous chemical histone modifications, affecting histone tails together or sequentially, modulate chromatin structure and thus regulate gene expression [11].

It was demonstrated that, despite only 2% of human transcripts being encoding proteins, more than 70% of the human genome is transcribed in non-coding RNAs (ncRNAs) that are therefore the biggest part of the genome [12,13]. NcRNAs can be divided into housekeeping (ribosomal RNAs (rRNAs), transfer RNAs (tRNAs), small nuclear RNAs (snRNAs) and small nucleolar RNAs (snoRNAs)) and regulatory ncRNAs, whereas the last category is commonly further subdivided into small ncRNAs (small interfering RNAs (siRNAs), microRNAs (miRNAs) and piwi-interacting RNAs (piRNAs)) and long non-coding RNAs (lncRNAs), depending on their length. Their multiplicity, conservation and active transcription further suggest their functional importance and make them valuable molecular candidates for diagnostic and therapeutic approaches for diseases [2]. Abnormal expression of ncRNAs has been reported in many pathological states including cancer, type II diabetes, rheumatoid arthritis and cardiovascular and neurodegenerative diseases [14].

A group of small ncRNAs includes transcripts of 15–200 nucleotides (nt) participating in the genome defense and post-transcriptional regulation of gene expression. Up to now, the role of small ncRNAs was reported in almost all central processes, such as maintenance of stemness and germline, development and differentiation and transcriptional and post-transcriptional gene silencing [15].

MiRNAs are the most intensively studied class of small ncRNAs. They comprise evolutionary conserved transcripts of 17–25 nt that regulate gene expression on the post-transcriptional level by either repressing the translation of their target mRNAs or causing mRNA degradation [16]. The mechanism is determined by the degree of complementarity between the mRNA 3′ untranslated region (UTR) of its target mRNA and nucleotides positioned 2–7 from the 5′ end of miRNA called the “seed sequence” [17]. MiRNAs that are completely complementary with their mRNA targets can directly cleave and degrade them. However, they are very rare, whereas most miRNAs target mRNAs are partially complementary and lead to translational repression [1,18]. SiRNAs are also derived from long double-stranded RNA molecules that are processed by the Dicer enzyme. The resulting 19–24 nt transcripts are loaded onto Argonaute proteins and can lead to transcriptional gene silencing in cells [1,19]. In contrast to the two previous classes of small ncRNAs, piRNAs come from a single chain precursor instead of a double-stranded RNA, and its biogenesis does not depend on the Dicer enzyme [20]. piRNAs are 26–31 nt in length and are bound to Piwi proteins that are involved in epigenetic regulation (55,56). The Piwi proteins bind to genomic PcG response elements together with PcGs and thus silence the homeobox genes [21].

LncRNAs are the most diverse class of non-coding RNAs. They are over 200 nucleotides in length, located in the nucleus or cytoplasm, rarely encode proteins, are less conservative among species and usually demonstrate cell- and tissue-specific expression patterns. A number of studies confirmed the important role of lncRNAs in the regulation of transcription, translation, splicing, cell growth and differentiation, apoptosis, cell cycle, dosage compensation, imprinting, pluripotency and control of chromatin structure and modifications [1,22,23].

LncRNAs may have features similar to mRNAs of protein-coding genes. Thus, some of them have a short open reading frame (ORF) and polyadenylation at the 3′ end and are transcribed by RNA polymerase II. They have exons, although shorter and less numerous than in protein-coding genes, and therefore can undergo splicing and form isoforms with different functions. However, they also can undergo alternative 3′ processing, which gives them a variety of structures [22]. Around 60% of mammalian lncRNAs are transcribed from the same DNA regions as protein-coding genes, sometimes from a common promoter, and as a result are mutually regulated [24]. LncRNAs are further subdivided depending on the genomic localization of lncRNA genes and their positioning relative to protein-coding genes into groups of the intergenic, intronic, antisense, bidirectional and overlapping lncRNAs [22]. As a result of noncanonical splicing, intronic and overlapping sense lncRNAs can produce circular forms (circRNAs), which are pervasively expressed in mammalian tissues, highly stable and also could act as transcriptional and translational regulators [25]. There is also a specific subgroup of macroRNAs that is several hundred kb long and could include multigene transcripts or even a whole chromosome [26].

In contrast to small ncRNAs, lncRNAs can regulate gene expression on both transcription and post-transcriptional levels and through a variety of mechanisms [27]. The reasons for its diversity are not only the already-mentioned localization in different subcellular compartments and different structure but also their ability to form secondary and tertiary structures, which enables them to interact with proteins and chromatin [28]. Most lncRNAs act in complexes with proteins. In such complexes, lncRNAs can act as scaffolds, guides, decoys and signals. Molecular scaffolds assemble proteins into complexes and initiate various biological processes; guides direct proteins to the specific sites of the genome; decoys bind proteins and prevent their interaction with target genes, whereas signals mark specific loci and developmental stages, to regulate transcription [22]. LncRNAs can also act separately, as ribozymes or riboswitches. Through multiple specific binding sites within the lncRNA sequences, they can bind miRNAs and prevent their functioning, acting as miRNA sponges [29].

The organization and packaging of DNA into chromatin are mediated by mutual interaction between all epigenetic regulators. Working together, DNA methylation, histone modifications and ncRNAs determine the specific structure of chromatin and create stable and clear patterns of gene expression [30].

The specific organization of DNA into chromatin in the cell nucleus may reflect both the normal state of the cell and the presence of pathological processes. Epigenetic modifications are better investigated in carcinogenesis, partially because of the availability of somatic tumor tissues that can be obtained during surgical treatment of patients. The study of epigenetic mechanisms in tumor tissues enables determination of their associations with the clinical characteristics of patients and, as a result, helps to propose them as clinical and prognostic markers. Currently, an accumulation of novel data about the role of epigenetic changes in other pathological processes is also increased [31].

In our review, we summarize the current literature data on the epigenetic processes both in normal and pathological conditions. The recent investigations have confirmed that epigenetic regulators of different types act in cells as a complex regulatory network. As DNA methylation has been known for a longer time and is better investigated, we are focused on another two groups of epigenetic regulators, namely histone modifications and ncRNAs. For this point, we highlight some recently described examples of ncRNAs and histone marks and discuss possible mechanisms of their interaction. Furthermore, we analyze their role in the development and pathogenesis of diseases and their application in diagnosis and therapy, including the currently approved epi-drugs and biomarkers and ongoing clinical trials. Finally, we briefly cite the advantages and knowledge gaps of epigenetics.

## 2. Histone Modifications and ncRNAs: The Theory

In a eukaryotic cell, DNA is wrapped around nucleosomes—octamers of histone core proteins, consisting of two of each H2A, H2B, H3 and H4—thus forming chromatin. The N-term tails of histone core proteins are enriched with arginine and lysine residues and undergo covalent post-transcriptional modifications. This structure of chromatin together with histone modifications determines the ability of interaction between DNA and transcription factors [23]. Depending on the histone type, amino acid residue and chemical functional group (methyl group (methylation), acetyl group (acetylation), phosphate group (phosphorylation), ubiquitin (ubiquitinylation), SUMO (sumoylation), hydrophobic isoprene polymers (isoprenylation), glycosyl group (glycosylation), etc.) histone modifications activate or repress the transcription of genes [32,33] (Table 1). Besides, they can demonstrate different degrees of modification (mono-, di- and trimethylation), localization (gene body, promoter and CpG island) and pattern of modification [34].

Histone modifications are localized at different sites and form a specific histone modification signature that predicts gene expression patterns and dynamically changes during cellular processes, including cell differentiation, regeneration and diseases [35]. For example, in cardiac development, cardiomyocytes show mono-methylation of lysine 4 on histone H3 (H3K4me1) during early stages but trimethylation H3K4me3 at later stages [36]. The genes coding adult isoforms of proteins α-myosin heavy chain (α-MHC) and the transcription factor NKX2.5 are characterized by high levels of H3K27me3 deposition at the pluripotent stage but are gradually replaced by H3K4me3 modification [37].

Histone modifications of different types can also affect each other in crosstalk. For example, the phosphorylation of serine 10 on histone H3 (H3S10ph) inhibits trimethylation of lysine 9 on histone H3 (H3K9me3) [38] and promotes acetylation of lysine 16 on histone H4 (H4K16ac) [39]. Disbalanced activation/inactivation of the modifications could change programs of gene expression and lead to a pathogenesis associated with transcriptome aberrations. Thus, almost any mutation in genes involved in histone modifications may contribute to the development of diseases, including cancers, autoimmune and neurodegenerative disorders [40].

**Figure 1:**
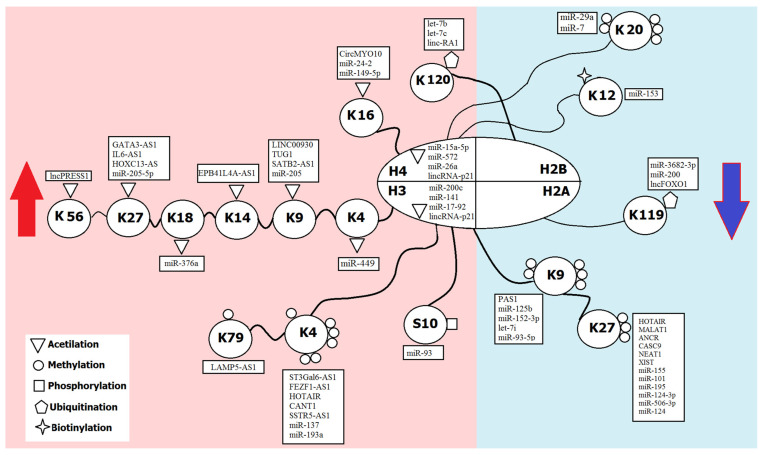
Regulation of histone modifications by ncRNAs. H3, H4, H2B, H2A—histone proteins; K—lysine residue; S—serine residue. The part of the figure with the repressive histone marks is highlighted in blue; the part with the activating histone marks is highlighted in red.

NcRNAs are tightly associated with regulation of deposition, alterations and removal of histone marks both in normal and pathological conditions. MiRNAs are one of the largest classes of epigenetic regulators with 2 654 mature human miRNAs described (miRbase, release 22.1, October 2018) [41] that are expressed in virtually all tissues at all stages of development, and almost all of them can target numerous transcripts in all types of cells. This multiplicity of target genes is provided by the ability of miRNAs to interact with mRNAs in their recognition site of the “seed sequence”, which is only 6 nt in length and is not necessary to be perfectly complementary [2]. Among direct targets of miRNAs are genes that code protein complexes and enzymes involved in the histone modification processes, such as HMTs, HDACs and polycomb genes [10]. The involvement of siRNA-induced transcriptional gene silencing in histone modifications was also confirmed in several studies [42]. LncRNAs are even more numerous. For example, the NONCODE database has estimated the number of human lncRNA transcripts as 173 112 [43]. Besides, they can regulate gene expression in different cell compartments and by variable mechanisms. LncRNAs are able to interact with protein complexes, referred to as histone writers, erasers and readers of histone modifications, thus recruiting them to certain subnuclear domains and genetic loci [44]. They serve as a signal for decoding chromatin modifications and act as molecular scaffolds for specific chromatin regulatory complexes that combine and integrate the molecular functions of several histone modifiers, thus adjusting the chromatin structure to a specific target locus [45]. Being able to sponge miRNAs, lncRNAs are often regulating histone modification via their target miRNAs, forming regulatory axes. As a result, ncRNAs regulate the addition to amino acid residues of histones of almost all chemical functional groups, their number and localization (Figure 1).

**Figure 2:**
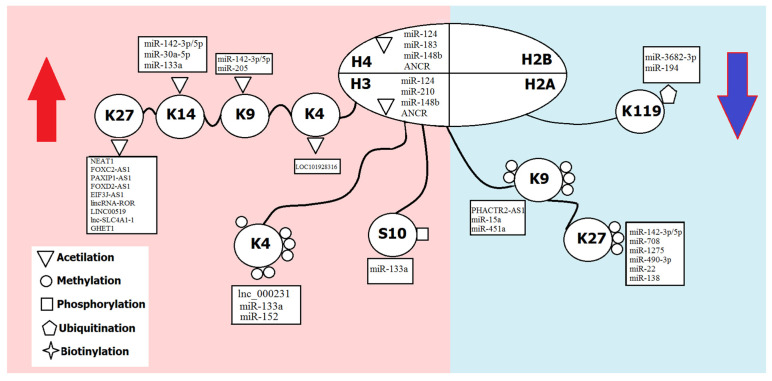
Regulation of ncRNAs by histone modifications. H3, H4, H2B, H2A—histone proteins; K—lysine residue; S—serine residue. The part of the figure with the repression of ncRNA expression is highlighted in blue; the part with the activation of ncRNAs is highlighted in red.

Alternatively, both miRNA and lncRNAs are also regulated through histone modifications, such as methylation and HDAC overexpression. Enzymes and components of the protein complexes involved in histone modifications can be recruited to the promoter regions of ncRNA genes and thus activate or repress their expression [46] (Figure 2).

Therefore, interactions between non-coding RNAs and histone modifications resemble rather complex regulatory networks. Most non-coding transcripts are described as regulators of specific histone marks. However, some ncRNAs are able to regulate several types of them simultaneously because of their targets. In the last decade, numerous non-coding transcripts were confirmed as regulators of histone modifications (Table 2), and their number is still expanding.

### 2.1. Histone Methylation and Demethylation

In contrast to DNA methylation, which is associated with transcription repression, histone methylation can either repress or activate gene expression, depending on the residue that is methylated, region of the gene and level of the modification [162]. Histone methylation occurs at lysine (K) and arginine (R) residues on histones H3 and H4 with an addition of either one (me1), two (me2) or three (me3) methyl groups. The activation marks are H3K4me1, H3K4me2, H3K36me2, H3K4me3 and H3K36me3, whereas H3K9me3, H4K20me3 and H3K27me3 are considered repressive [163].

The balance of histone methylation is provided by the collaboration between histone methyltransferases (HMTs) and histone demethylases (KDMs). Histone methylation and demethylation are the result of several protein complex activities. The histone-lysine N-methyltransferase 2 (KMT2) family proteins, namely MLL1 (MLL/KMT2A), MLL2 (KMT2B), MLL3 (KMT2C), MLL4 (KMT2D), SETD1A (KMT2F) and SETD1B (KMT2G) provide histone-lysine methyltransferase activity embedded within the COMPASS (complex of proteins associated with SET1). COMPASS are large multi-subunit protein complexes that catalyze di- and trimethylation of histone H3 lysine at positions 4 (H3K4) and are important for RNA-dependent polymerase II transcription in mammals [164]. In the promoter and enhancer regions of the genes, methyl groups are transferred to the N-terminal tails of H3 in nucleosomes and are universally associated with transcription activation. SET1A/B complexes are involved in H3K4 mono- and dimethylation throughout the whole genome, whereas KMT2A/B complexes (MLX) are restricted in targets of its enzymatic activity. They catalyze H3K4 di- and trimethylation at nearby gene promoters and cis-regulated sites, where they maintain active transcription [165]. MLR COMPASS-like complexes contain KMT2C and KMT2D monomethylate lysine H3K4 (H3K4me1) in transcription enhancers throughout the human genome at from 12,000 to more than 20,000 sites, depending on cell type and development stage [166].

Polycomb group proteins (PcG) are also involved in the methylation of histone tails. PcG complexes include polycomb repressive complexes 1 and 2 (PRC1 and PRC2) and the recently identified Pho-repressive complex and repressive deubiquitinase polycomb [167]. PRCs demonstrate a histone-modifying activity, which promotes transcription repression. PRC1 monoubiquitinylates a histone H2A lysine at position 119 (H2AK119ub1) and can compact the chromatin structure by binding to nucleosomes, whereas PRC2 is a lysine methyltransferase (KMT) that trimethylates histone H3 lysine at position 27 (H3K27me3), which is associated with transcription repression. The PRC1 and PRC2 complexes can function simultaneously to epigenetically block gene expression [168,169].

PRC2 consists of several subunits, namely embryonic ectoderm development (EED), suppressor of zeste 12 (SUZ12) and enhancer of zeste homolog 1 or 2 (EZH1 or EZH2), which has a conservative catalytic domain SET. The previous investigation revealed that approximately 24% of all human lncRNAs can physically bind to EZH2 and recruit it to the target genes [170].

Histone demethylases (HDMTases) are divided into the amine-oxidase type lysine-specific demethylases (LSDs) and JumonjiC (JMJC) domain-containing histone demethylases, which mediate the demethylation of specific regions. LSDs include LSD1 (KDM1A) and LSD2 (KDM1B), which specifically target H3K4me1 and H3K4me2. In addition, KDM1A was also found to demethylate at H3K9me1 and H3K9me2 [163]. JMJC domain-containing histone demethylases are the highly conserved group that consists of 20 families, including JHDM1, JHDM2 (JMJD1), JHMD3 (JMJD2), JARID, PHF and UT families [171].

An arginine methylation is regulated by nine protein arginine methyltransferases (PRMTs), subdivided into three classes based on the type of methylation. The type I PRMTs (PRMT1, PRMT2, PRMT3, PRMT4, PRMT6, PRMT8) generate asymmetric dimethylarginine; type II PRMTs (PRMT5 and PRMT7) form symmetric dimethylarginine (SDMA); and type III PRMTs (PRMT7) provide monomethylarginine, whereas PRMT9 is beyond all classes. PRMTs are involved in mRNA splicing, X chromosome inactivation, DNA repair and tumorigenesis [172].

The interactions of histone methylations with ncRNAs were already investigated in a number of studies, and their deregulations are commonly associated with diseases. LncRNAs regulate histone methylations mostly by interacting with histone methyltransferases and demethylases or recruiting chromatin modifying complexes, such as PRC2 and COMPASS-like complexes, whereas miRNAs target the mRNAs of the proteins involved in processes of histone methylation [173]. For example, lncRNA PHACTR2-AS1 (PAS1) forms a complex with the RNA-binding protein vigilin and histone methyltransferase SUV39H1. The recruitment of SUV39H1 triggers H3K9 methylation of human hyaluronidase family member PH20 and inhibits breast cancer growth and metastasis [103]. Being overexpressed in breast cancer tissues, lncRNA ROR promotes breast cancer progression via repression of transmethylase mixed-lineage leukemia 1 (MLL1) and tissue inhibitors of metalloproteinase 3 (TIMP3) [72] Dual-luciferase report assay and investigation of both cell lines and samples of patients with acute lymphoblastic leukemia confirmed that miR-137 directly targets lysine-specific demethylase JARID1B mRNA, leading to abnormal levels of H3K4me3 and H3K4me2 [86].

One of the most investigated lncRNAs, antisense non-coding RNA in the INK4 locus (ANRIL), also known as CDKN2B-AS1, is among the five genes located in the 9p21 locus. This chromosomal region is a hotspot for mutations that are associated with the development of various pathologies, particularly with tumors and cardiovascular diseases [174]. ANRIL expression and its splicing isoforms are associated with such diseases as myocardial infarction, atherosclerosis, calcifying aortic stenosis, type 2 diabetes, glaucoma and endometriosis [175]. ANRIL acts mainly *in cis*, mediating epigenetic silence of tumor suppressor genes *CDKN2A* and *CDKN2B*. It has been shown that ANRIL interacts with proteins of the PRC1 and PRC2, which leads to the deposition of repressive H3K27me3 marks on the target genes *CDKN2A* and *CDKN2B* through mechanisms of histone modification and chromatin remodeling. ANRIL interacts with both PRC1 and PRC2, mediating epigenetic transcription repression of neighboring genes. Thus, ANRIL interacts with a component of the PRC1–CBX7 complex to recruit PRC1 to the p14ARF and p16INK4a loci suppressing the CDKN2A locus using H3K27-trimethylation [176]. For CDKN2B, ANRIL recruits SUZ12, a subunit of PRC2 [174]. ANRIL also interacts with the PRC-associated protein YY1, which is involved in establishing the conformation of the three-dimensional genome [177].

Even better known is the *HOX* transcript antisense RNA (HOTAIR), which was already described as a potent oncogene in many tumors and plays an important role in many physiological and pathological processes in the cell. It is involved in the epigenetic regulation of histones by interacting with protein complexes PRC2 and LSD1/COREST [60]. The binding of HOTAIR with EZH2 can enhance H3K27me3 to regulate many related genes [163]. Thus, HOTAIR promotes epithelial–mesenchymal transition (EMT) in gastric cancer by switching H3K27 acetylation to methylation at the E-cadherin promoter and inhibiting its expression [61]. HOTAIR suppress p15 expression through EZH2-enrolled H3K27me3 in p15 promoter and thus promotes the self-renewal of leukemia stem cells [62]. Upregulating histone H3K27 demethylase JMJD3 and regulating the expression of methyltransferase EZH2 target gene *PCDHB5*, HOTAIR plays a dual regulatory role in chromatin state by affecting both histone methylation and demethylation at different gene loci and promotes metastasis of renal cell carcinoma [63].

Antisense transcript 1 *ST3Gal6* (ST3Gal6-AS1) is transcribed from the promoter region of the *ST3Gal6*. Bioinformatic analysis and experiments have shown that lncRNA ST3Gal6-AS1 binds histone-methyltransferase MLL1, recruits it to the *ST3Gal6* promoter region and induces H3K4me3 modification and subsequently activates *ST3Gal6* transcription. ST3Gal6-AS1 and *ST3Gal6* were found to be suppressed in colorectal carcinoma cells, and their expression levels were positively correlated. H3K4me3 signals were detected in the *ST3Gal6* promoter region, whereas ST3Gal6-AS1 knockdown affects H3K4me3 accumulation [112].

FEZ family zinc finger 1 antisense RNA 1 (FEZF1-AS1) was recently discovered as involved in proliferation, apoptosis, migration and invasion of various malignant tumors [53]. FEZF1-AS1 is 2 564 bp long, localized on chromosome 7 and regulates *FEZF1*, which is involved in the pathogenesis of colorectal carcinoma and glioma and acts as an activation factor of the signaling pathways Wnt and Akt-ERK in a tumor [54]. Besides, FEZF1-AS1 participates in P21, H3K4me2 and LSD1 regulation. It was experimentally confirmed that lincRNA FEZF1-AS1 specifically binds LSD1, which demethylates mono- and dimethylated H3K4, thus regulating the expression of the gene encoding the cyclin-dependent kinase inhibitor CDKN1A (P21) at the transcription level. FEZF1-AS1 knockdown also affects H3K4me2 modification and the bond between LSD1 and P21 promoter. Transcription factor SP1 activates the FEZF1-AS1 expression by binding to its promoter [55]. Overexpression of FEZF1-AS1 has been determined in various types of tumors, where it is associated with tumor cell growth and hyperproliferation because of cell cycle progression and a decrease of apoptosis [178].

A significant number of ncRNAs were confirmed to regulate methylation/demethylation processes by interacting with EZH2. For example, lncRNA SNHG7 (small nucleolar RNA host gene 1) acts as an oncogenic in cervical cancer by recruiting EZH2 to be promoter of the inhibitor of the Wnt/β-catenin signaling *DKK*, therefore epigenetically enhancing the Wnt/β-catenin signaling [109]. Interacting with the EZH2, another member of this family, SNHG8 inhibits the expression of tumor suppressor reversion-inducing cysteine-rich protein with kazal motifs (RECK) at the transcriptional level, promoting cervical cancer [110]. SNHG1 inhibits the osteogenic differentiation of periodontal ligament stem cells through EZH2-mediated H3K27me3 methylation of *KLF2* promotor [106] and also promotes bladder cancer progression by interacting with miR-143-3p as an endogenous sponge and EZH2 [107]. LncRNA X-inactive specific transcript (XIST) was found to be significantly upregulated in neuroblastoma tissues and cell lines. Additionally, its effect on cell growth, invasion and migration was mediated by interaction with EZH2 and subsequent increase of H3K27me3 level in *DKK1* promoter [115].

MiRNAs could also inhibit EZH2. Thus, miR-101 and miR-195-5p inhibit proliferation and promote apoptosis in laryngeal squamous cell carcinoma [80] and in gestational diabetes mellitus [179], respectively. In both studies, the miRNAs were predicted to directly target *EZH2* and were assessed using a dual luciferase reporter assay. *EZH2* was confirmed as a direct target for miR-214 and miR-137, which act as tumor-suppressors in cervical cancer [92]. Their low level in a tumor is correlated with the poor prognosis and short-term survival of patients. However, being upregulated, they suppress *EZH2* expression and subsequently suppress the cell proliferation [180].

EZH2 could be not only a target for miRNAs but also an upstream regulator. Chang and Zhou revealed that miR-22-3p is regulated by EZH2 in synovial tissues and fibroblasts-like synoviocytes from patients with rheumatoid arthritis. They demonstrated that EZH2 can bind to the miR-22-3p promoter and increase the H3K27me3 methylation level, therefore downregulating the miRNA and increasing expression of its target gene *CYR61*, thus stimulating proliferation, migration and invasion of fibroblast-like synoviocytes from patients with rheumatoid arthritis [181]. The suppression of miRNAs by EZH2 via CpG methylation was confirmed for miR-484. EZH2 mediates the suppression of miR-484 expression in cervical cancer by recruiting DNMT1 to its promoter [182]. EZH2-determined H3K27me3 enrichment at the miR-138 promoter region suppresses miR-138 expression and upregulates its target gene *SDC1*, promoting cartilage degeneration in osteoarthritis [183].

Other proteins involved in histone methylation and demethylation are also confirmed as subjects of ncRNA regulation. MiR-199b-3p protects renal tubules from diabetic-induced injury by repressing KDM6A, a histone lysine demethylase regulating E-cadherin expression. Overexpression of hsa-miR-199b-3p increases E-cadherin expression and prevents EMT through repressing KDM6A expression in HG-induced HK2 cells. In contrast, inhibitor-induced hsa-miR-199b-3p knockdown has opposite effects, as it decreases E-cadherin level and worsens EMT, accompanied by increased levels of KDM6A [91]. KDM2A physically interacts with the promoter of miR-132 and suppresses its expression by removing the mono- or dimethyl group from H3K36 at the miR-132 locus. This results in further activation of the miR-132 target gene radixin (*RDX*) and promotes cervical cancer progression [184].

MiR24-2 promotes the proliferation in human liver cancer stem cells by targeting the 3′ UTR of *PRMT7*. Suppression of PRMT7 leads to inhibition of the di-/trimethylation of histone H4 arginine 3 (H4R3) and promotion of *Nanog* expression via long non-coding RNA HULC [94]. Conversely, PRMT3 activates the expression of miR-3648 by enhancing histone H4 arginine 3 asymmetric dimethylation (H4R3me2a) levels at the promoter region of the gene. Overexpression of miR-3648 rescues impaired osteogenesis in PRMT3-deficient cells [185].

The effect on histone proteins could have an important influence on the telomeres as well. Thus, it was determined that lncRNA maternally expressed gene3 (MEG3) blocks telomerase activity in human liver cancer stem cells, acting in several ways simultaneously. MEG3 enhances the *P53* expression and thus increases the methylation modification H3K27me3 in telomerase reverse transcriptase (*TERT)* promoter regions. Besides, MEG3 increases the expression of the telomeric repeat-containing RNA (TERRA) [186].

Although lncRNAs and miRNAs are more commonly described as participants of mutual regulation with histone modifications, there are several studies devoted to ncRNAs from other classes. Zhou et al. conducted experiments in human non-small cell lung cancer and gastric cancer cells A549/DDP and AGS/DDP and demonstrated that siRNAs could suppress *EZH2* and thus reverse cisplatin resistance [187]. Among the ncRNAs tightly connected with EZH2 expression are also circRNAs. They are produced in various post-transcriptional processes and were found to be upregulated in many types of cancer [188]. CircAGFG1 contributes to tumor progression by recruiting EZH2 to the promoter of tumor suppressor p53 and inhibiting its expression [51]. Circ_0019435 recruited EZH2 by directly binding to silence the expression of *DKK1* and *PTEN*, thereby enhancing the progression of cervical cancer [50]. CircRNAs can also target other components of the histone methylation system. Circ_SPECC1 acts as a sponge to bind miR-526b and therefore regulates its target genes, including lysine demethylase 4A (*KDM4A*) and the downstream signaling target *YAP1/KDM4A*, inhibiting the invasion and growth of gastric cancer cells [189]. PiRNAs regulate trimethylation of H3K9, which is an important controller of initiation, propagation and maintenance of heterochromatin [190]. Pezic et al. proposed the model for piRNA-induced establishment of the H3K9me3 mark on L1 elements and demonstrated that, in addition to DNA methylation, the piRNA pathway is required to maintain a high level of the repressive H3K9me3 histone modification on long interspersed nuclear elements (LINEs) in germ cells [191].

### 2.2. Histone Acetylation and Deacetylation

Histone acetylation/deacetylation is a reversible process of attaching or removing an acetyl group to histone proteins, catalyzed by histone acetyltransferases (HAT) or histone deacetylases (HDAC), respectively. Currently, it is known that not only histones but other proteins also can be subjects of acetylation and deacetylation processes. Investigations of acetylome have allowed for the discovery of many non-histone substrates, such as AML1, p53, c-MYC, NF-kB, cohesin and tubulin, which play an important role in various cellular processes [192]. Histone acetylation is associated with transcription activation, whereas histone deacetylation inhibits gene expression. The main sites of histone acetylation are H3K4, H3K9, H3K27, H3K56 and H4K16. It is considered that the addition of an acetyl group to the α-amino group on the lysine residue of a histone neutralizes the positive charge of lysine. As a result, the bonds between DNA and histones are weakened following chromatin decondensation and transcription activation [193]. Histone proteins have structural and functional roles almost in all nuclear processes, and its aberrations are associated with such diseases as tumors, liver injury, viral hepatitis and Alzheimer’s disease [23,194].

Depending on the cellular localization, HATs are divided into two groups. The type A HATs have a nuclear localization and are associated with transcriptional acetylation of histones in chromatin, whereas type B HATs are localized in the cytoplasm, acetylate newly synthesized histones and affect the structure of nucleosomes [195]. In humans, there are about 30 HATs that are divided into three families: the general control non-depressible 5 (GCN5)-related N-acetyltransferase (GNAT) family (GCN5 and p300/CBP-associated factor (PCAF)), the MYST family (monocytic leukemic zinc factor (MOZ), MOZ-related factor (MOF), Tat interactive protein 60kDa (TIP60) and human acetylase binding to PRC1 (HBO1)) and the p300/CBP family (p300 and CBP). Besides, there are many other nuclear receptors and transcription factors in addition to these HATs [171].

Transcription coactivators of CREB-binding protein (CBP) and p300 are highly homologous acetyltransferases that mediate acetylation of histone 3 lysine 27 (H3K27ac) in regulatory elements such as enhancers and promoters. Deregulations of HATs are associated with pathologies, including carcinogenesis. The p300 and CBP regulate several fundamental biological processes, such as proliferation, cell cycle, cell differentiation and DNA damage response [196].

Acetylated lysine in histones can bind to certain epigenetic readers, namely the bromodomains and extraterminal (BET) protein family (BRD2, BRD3, BRD4 and BRDT). The BET proteins regulate gene transcription via bromodomains by binding to acetylated lysine residues on histones and transcriptional factors [197]. The human genome encodes more than 60 bromodomain proteins, including HAT, HAT-associated GCN5L2, PCAF and BRD9 proteins, histone methyltransferases (ASH1L and MLL), transcription coactivators (TRIMs and TAF), as well as BET family proteins [198]. BRD4, a member of the BET protein family, is localized in the enhancer and/or promoter regions of many active genes. Bromodomains serve as regulators of protein–protein interaction in cellular processes such as transcription and chromatin remodeling. They are also present in 46 different types of proteins, such as transcription coactivators, chromatin-associated proteins and methyl- and acetyltransferases [197]. BET proteins can bind to acetylated histone lysines located in the DNA regions enriched with repressive marks H3K27 and RNA polymerase II (super-enhancers). After the binding, BET proteins participate in the recruitment of P-TEFb to the transcription complex via the C-terminal domain (CTD), thus forming a heterodimer of CDK9 and cyclin T1 or T2. It determines the degree of chromatin compaction, acting as a regulator of gene expression [197].

HDACs are post-transcriptional modulators that remove acetyl groups from lysine residues of both histone and non-histone proteins [199]. In humans, 18 HDACs are grouped into four classes according to their structural homology: class I (HDAC 1, 2, 3 and 8); class II (HDAC 4, 5, 6, 7, 9 and 10); class III (SIRT 1, 2, 3, 4, 5, 6 and 7) and class IV (HDAC 11). HDACs form complexes with other enzymes and can cross-interact with other epigenetic factors in the regulation of cellular functions in normal and pathological conditions. HDAC 1 and 2 are expressed in all tissues and are involved in the processes of proliferation and apoptosis, while HDAC 3 is involved in the formation of a response to DNA damage. HDACs 4, 5, 7 and 9 are associated with cell differentiation and development. Sirtuins are mainly involved in cellular metabolism and DNA repair, and HDAC 11 is involved in the regulation of interleukin expression [200,201,202].

LncRNAs regulate histone acetylation/deacetylation by a similar mechanism as in histone methylation. LncRNAs recruit to certain loci histone acetyltransferases or deacetylases, such as HDAC, HAT, CBP/P300, KAT2A and SIRT6, thus affecting gene transcription [203]. LncRNA P53-regulated and ESC-associated 1 (lncPRESS1) is regulated by p53 and is necessary for the maintenance of embryonic stem cell pluripotency. Acting as a sponge, lncPRESS1 binds nuclear histone deacetylase SIRT6, which erases histone marks H3K56ac and H3K9ac in embryonic stem cells [204]. It is proposed that lncPRESS1 isolates SIRT6 from the loci of the genes, therefore maintaining their high acetylation level. During differentiation, lncPRESS1 expression decreases and SIRT6 is recruited to these genomic loci, following the deacetylation of H3K56 and H3K9ac and loss of pluripotency [133]. Already mentioned, lncRNA ANRIL is associated with the development of cardiovascular diseases. It is overexpressed in patients with coronary atherosclerotic heart disease, and at the same time its downregulation by siRNA results in a decrease of the reactive oxygen species (ROS) level and the phenotypic changes in human aortic smooth muscle cells. The search of the mechanism of ANRIL action revealed its ability to act as a scaffold, binding WD repeat-containing protein 5 (WDR5) and HDAC3 and providing their connection to NADPH oxidase 1 (*NOX1)* promoter, which could be the reason for such alterations in the smooth muscle cell [118]. Besides, ANRIL directly recruits PRC1 and PRC2 to modify histones and thus regulate hundreds of genes *in cis* regulation [117].

LncRNA nuclear paraspeckle assembly transcript 1 (NEAT1) is abnormally expressed in numerous tumors and functions as an oncogene. It is also upregulated in laryngocarcinoma tissues and cells and promotes cell proliferation and metastasis through miR-524-5p. Acting as a sponge, NEAT1 inhibits miR-524-5p expression and subsequently increases the expression of HDAC1 and acetylation of PTEN and AKT [150]. Conversely, H3K27 acetylation on the NEAT1 promoter starts the regulatory pathway miR-212-5p/*GRIA3* and promotes hepatic lipid accumulation in non-alcoholic fatty liver disease [149]. NEAT1 also interacts with p300 and regulates IL-18 expression by acetylating histones in the IL-18 promoter region, which leads to a worse clinical picture of rheumatoid arthritis [152]. Overexpression of NEAT1 is also associated with several cognitive and neurodegenerative disorders, such as Parkinson’s disease, Alzheimer’s disease, Huntington’s disease and schizophrenia. Studies on humans and rodents have confirmed the important role of NEAT1 in neuroplasticity [100]. Alzheimer’s disease is characterized by accumulation of the intracellular β-amyloid peptide. NEAT1 binds to the P300/CBP complex, affecting also the acetylation of H3K27 (H3K27ac) and crotonylation of H3K27 (H3K27Cro) near the transcriptional start site of many genes, including genes associated with endocytosis. NEAT1 also mediates binding of STAT3 and H3K27ac but not H3K27Cro. Therefore, a H3K27ac decrease and H3K27Cro increase upregulate many genes involved in Alzheimer’s disease development [151].

Age-related macular degeneration appears because of scarring and vision loss. It is mediated by choroidal neovascularization, promoted by the sustained retinal hypoxia in retinal pigment epithelium cells. LncRNA antisense RNA of histone deacetylase 4 (HDAC4-AS1) was overexpressed in hypoxia compared to normal conditions, whereas HDAC4 expression was decreased in the hypoxic state. The HDAC4-AS1 transcript binds to the *HDAC4* promoter and promotes HIF-1α, thus inhibiting its expression [205].

LncRNA metastasis-associated lung adenocarcinoma transcript 1 (MALAT1) was confirmed as a regulator of histone methylation, but it can also regulate histone acetylation. MALAT1 induces the dysfunction of pancreatic β-cells via reducing the H3 histone acetylation of the pancreatic and duodenal homeobox 1 (*PDX-1)* promoter. Inhibition of the PDX-1 expression suppress the insulin secretion [134]. HOTAIR is also the lncRNA that regulates both histone methylation and acetylation. In exosomes, it promotes proliferation, invasion, migration and inhibits the apoptosis of endometrial stromal cells. Acting as a competing endogenous RNA, it downregulates miR-761 and increase expression of its target gene *HDAC1* [126]. It also plays an important role in EMT regulation in gastric cancer, switching the acetylation of H3K27 to methylation by the E cadherin promoter, which induces inhibition of E-cadherin transcription [61].

The interleukin 6 antisense RNA 1 (IL6-AS1), which is upregulated in chronic obstructive pulmonary disease, was found to be expressed both in the nucleus and cytoplasm compartments. Cytoplasmic form of IL6-AS1 acts as an endogenous sponge for miR-149-5p and stabilizes IL-6 mRNA. Nuclear IL6-AS1 recruits early B-cell factor 1 to the IL-6 promoter, thus increasing the methylation of the H3K4 and acetylation of the H3K27 and promoting IL-6 transcription [64].

MiRNAs also regulate histone acetylation/deacetylation by the same mechanism as histone methylation; the difference is only in target genes and their functions. A hypermethylation of the tumor suppressor miR-589 gene promoter leads to the aberrantly high level of its target gene *HDAC5* and increases migration, invasion and tumorigenicity in non-small cell lung cancer [206]. Another miRNA, miR-520b, directly targets *HDAC4*, leading to a decrease of its expression both in lung cancer A549 cells and patient samples. The introduction of miR-520b was confirmed to inhibit cell proliferation in vitro, whereas anti-miR-520b accelerated lung cancer cell proliferation [145].

Rheumatoid arthritis is characterized by impaired fibroblast-like synoviocytes proliferation and a high release of inflammatory cytokines. MiR-138 can promote disease development by regulating inflammatory cytokines through its direct target HDAC4 following modulation of *PGRN* or *NF-κB* expression [207]. MiR-22 is a key regulator of stress-induced heart damage. It directly targets *SIRT1* by binding to the 3′ UTR and decreasing its expression. It was experimentally confirmed that application of miR-22 antagomirs upregulates SIRT1, increases resistance to oxidation in the heart and suppresses cardiomyocyte apoptosis and oxidative stress [139]. Another miRNA, miR-200a-3p, also involved in DOX-induced apoptosis and inflammation of cardiomyocytes, performs its function via indirect regulation of *SIRT1*. Targeting paternally expressed gene 3 *(PEG3*), miR-200a-3p mediates deacetylation of p53 and NF-κB p65 [208].

MiRNAs can also regulate the histone acetylation in so-called super-enhancers. Super-enhancers are clusters of transcription enhancers that drive gene expression, typically characterized by a high level of H3K27ac mark. In tumorigenesis, the pattern of histone modifications in such regions is frequently changed. Among the deregulated oncogenes is MYC, which is aberrantly expressed in many tumors. MiR-766-5p was confirmed to downregulate MYC expression by directly targeting *CBP* and *BRD4*. *CBP* suppression reduces levels of H3K27ac at MYC super-enhancers, whereas targeting of *BRD4* leads to a decrease of the BRD4-NUT fusion protein level. Therefore, miR-766-5p mediates the protumorigenic consequences of super-enhancers and oncogenic fusion proteins [146].

NcRNAs can form feedback loops with the HDACs, both regulating them and being regulated simultaneously. One of such feedback loops was described in patients with diabetic kidney disease and was confirmed in a mice model. Higher expression of HDAC2 in peripheral blood of these patients is partially related to the downregulation of miR-205, which directly targets *HDAC2*. At the same time, HDAC2 reduces histone H3K9 acetylation in the miR-205 promoter and inhibits its expression through an SP1-mediated pathway. miR-205 also regulates its own transcription by inhibiting *HDAC2* and increasing histone H3K9 acetylation in its promoter, forming a feedback regulatory loop [138]. Similarly, miR-376a and HDAC9 form a regulatory circuitry in hepatocellular carcinoma: HDAC9, which is a direct target of miR-376a, also increases the expression of miR-376a by upregulating the global histone H3K18 acetylation level [141].

Among epigenetic regulators of histone acetylation and deacetylation are several circRNAs. Chen et al. have demonstrated that circMYO10 sponges miR-370-3p and by its inhibition modulates the level of the RuvB like AAA ATPase 1 (RUVBL1) that can be complexed with chromatin remodeling, histone-modifying factor TIP60 and lymphoid enhancer factor-1 (LEF1) to promote histone H4K16 acetylation in the vicinity of the promoter region of gene *C-myc*. Therefore, CircMYO10 promotes osteosarcoma progression by regulating miR-370-3p [121]. CircITCH, which is produced by the reverse splicing of exons 7–14 of the itchy E3 ubiquitin protein ligase (*ITCH*) gene, acts as a tumor suppressor when sponging miR-330-5p and subsequently upregulates SIRT6, Survivin and SERCA2a. This regulatory axis modulates DNA damage, cell oxidative stress, cell death and contractile dysfunction in DOX-treated human-induced pluripotent stem-cell-derived cardiomyocytes [209]. CircMRPS35 suppresses gastric cancer development by recruiting histone acetyltransferase KAT7. KAT7 enriches H4K5 acetylation at the promoter regions of the forkhead box protein O1 (FOXO1) and forkhead box protein O3a (FOXO3a) and regulates expression of their downstream targets, either upregulating (p21, p27 and E-calmodulin) or downregulating (Twist1) [120].

### 2.3. Histone Phosphorylation and Dephosphorylation

H3 phosphorylation is a downstream event for a number of signal transduction pathways. The kinases ribosomal protein S6 kinase A3 (RSK2), mitogen- and stress-activated kinases 1 and 2 (MSK1/2), Pim-1 proto-oncogene, serine/threonine kinase (PIM1) and IκB kinase α (IKKα) have been shown to directly phosphorylate H3S10. As a target of diverse signaling cascades, H3 phosphorylation could be an important part of the transcriptional regulatory machinery [210].

In normal cells, histone mark H3S10ph is an additional modification that is involved in the formation of protein scaffolds, regulation of transcription, avoidance of repressive epigenetic information and prevention of heterochromatin formation in the loci. Histone phosphorylation affects the condensation and segregation of chromosomes. Deregulation of the H3S10 phosphorylation affects the functioning of kinetochores and divergence of chromosomes to the poles [211]. During cell division, the amount of the H3S10ph increases in contrast to the methylation of H3K9me2 or acetylation of H3K9ac [212]. Phosphorylation of H3S10 affects the formation of the chromatin structure and architecture, contributing to the displacement of the main heterochromatin protein HP1 [213]. Besides, H3S10ph participates in the formation of specific DNA–RNA hybrids called R-loops, which are formed during transcription. The resulting R-loops expose the DNA matrix strand, making it accessible to endonucleases and other enzymes that cause DNA damage. It contributes to the disruption of the chromosome structure and the appearance of chromosome instability [214].

H3S10 phosphorylation can be physically coupled to the acetylation of nearby lysine residues (K9 or K14); therefore, their combination is proposed to function together to activate transcription [210]. It was demonstrated that acetylation of lysines 9 and 14 of histone H3 increases their affinity to the protein family 14-3-3 that binds H3S10ph and thus protects them from dephosphorylation and stabilizes their expression [215]. The presence of H3S10ph (and H3K14ac) near H3K9me affects the binding of HP1 and causes its release from heterochromatin labeled by H3K9me [216]. In addition, H3S10ph affects the methylation of H3K4, making histone H3 more attractive for a complex with methyltransferase KTM2A/MLL1 and allows this complex to displace HP1- and polycomb-mediated condensed chromatin, promoting activation of transcription [217]. Many H3S10 kinases, including MSK1/2, PIM1, CDK8 and AURORA kinases, have long been considered as targets for cancer treatment [216]. Phosphorylation is a dynamic modification, and the H3 phosphorylation and dephosphorylation cycle linked to the activation and repression of genes, respectively, occurs within minutes to an hour. Despite the activation process being studied extensively, the mechanisms of H3 dephosphorylation are largely neglected [218].

There are only a few ncRNAs described as regulators of histone phosphorylation. miR-93 was characterized as an epigenetic switch in chromatin remodeling and structure formation in podocytes. MiR-93 targets a histone kinase *MSK2*, thus modulating its substrate H3S10. The investigation on the murine model confirmed a central role for miR-93 in diabetic nephropathy progression [158]. Investigation of the reversine effect in murine myoblasts revealed downregulation of miR-133a. It leads to a decrease in several active histone modifications, including trimethylation of histones H3K4 and H3K36, phosphorylation of H3S10 and acetylation of H3K14 on the miR-133a promoter, dissociation of RNA polymerase II from the promoter and an increase of the osteogenic lineage marker, Ogn, and the adipogenic lineage marker, ApoE expression [219].

### 2.4. Histone Ubiquitylation and Deubiquitylation

Histone ubiquitylation is a post-translational modification, characterized by the attaching of a small regulatory protein ubiquitin to the histone proteins. The name of a protein refers its ubiquity in all eukaryotic organisms [220].

Ubiquitin contains seven acceptor lysines (K6, K11, K27, K29, K33, K48 and K63), which are mostly located on histones H2A and H2B [220]. In mammalian systems, both H2A and H2B are ubiquitylated at K119 and K120, respectively [221]. Moreover, around 5–15% of total H2A and 1–10% of total H2B are ubiquitylated in higher eukaryotes [222]. Ubiquitylation regulates almost all processes in cells. Depending on the lysine and the configuration of the linkages, the ubiquitylation can drive different cellular outcomes. The addition of a ubiquitin molecule to histone results in monoubiquitination if conjugation appears at one of the lysines and multi-monoubiquitination when several lysines are ubiquitinylated [220]. Histone monoubiquitylation has a special role in DNA damage repair [223] and is associated with tumors and neurodevelopmental disorders; multi-monoubiquitylation has been mainly reported in endocytosis [220]. The ubiquitin can also form polymers and therefore provide the poly-ubiquitylation of proteins [224]. In this case, the addition of a single ubiquitin moiety attached at one or multiple lysine residues is the first step of poly-ubiquitylation. While H2A ubiquitylation mainly suppresses gene expression, H2B ubiquitylation enhances transcriptional activity. H2B ubiquitylation also influences chromatin remodeling and exhibits crosstalk with other histone modifications, such as trimethylation of H3K4 and H3K79 [225]. Similarly with the other histone modifications, histone ubiquitylation is a dynamic and therefore reversible process, and this is critical for the regulation of many biological processes by regulation gene transcription [226].

Histone ubiquitylation proceeds in three steps, involving an E1 ubiquitin-activating enzyme, an E2 ubiquitin-conjugating enzyme and an E3 ubiquitin ligase, which are required. In the first step, the E1 enzyme activates the C terminus of ubiquitin and forms a thiol ester of E1 and ubiquitin. Afterwards, the ubiquitin moiety is transferred from the E1 to the E2 enzyme, before being transferred to an appropriate substrate through the action of an E3 ligase. Being important for the specificity of substrate ubiquitylation, the E3 ligases determine the diversity of targets [225]. Deubiquitylation is catalyzed by ubiquitin proteases, also known as deubiquitylating enzymes or DUBs [227].

This and the next type of modification are less investigated than histone methylation or acetylation; however, several studies have also confirmed mutual regulation of ubiquitylation with ncRNAs. Thus, the let-7 family of microRNAs was confirmed to promote histone H2B ubiquitylation and inhibit cell migration by targeting multiple components of the H2B deubiquitylation machinery. The let-7 family includes many members and is frequently downregulated in cancer, acting as tumor-suppressors [228]. Monoubiquitylation of histone H2B (H2Bub1) is also reduced in cancer cells. Spolverini et al. demonstrated that let-7b and let-7c positively regulate cellular H2Bub1 levels by targeting multiple mRNAs, coding for distinct components of the H2B deubiquitylation machinery. Thus, let-7b and let-7c bind directly and inhibit the mRNAs encoding the DUBs ubiquitin-specific protease 42 and 44 (USP42 and USP44), the adapter protein ATXN7L3 and regulate the RNF20, which is the part of RNF20/RNF40 complex, catalyzing the monoubiquitylation of histone H2B [159]. Conversely, transcription of the miR-3682-3p gene is repressed by the monoubiquitination of histone H2AK119 after BMI1 is recruited on its promoter. The repression of miR-3682-3p activates P-glycoprotein and at least partially mediates chemoresistance of bladder cancer cells [229].

The radioresistance-associated long intergenic non-coding RNA 1 (linc-RA1), which is upregulated in radioresistant glioma cells and tissues, was found to stabilize the level of H2B K120 monoubiquitination (H2Bub1) by binding with H2B. Thus, it inhibits the interaction between H2Bub1 and ubiquitin protease USP44 and consequently suppresses autophagy, which is a key mechanism of radioresistance [160]. Another lncFOXO1 is associated with BRCA-1-associated protein 1 (BAP1) and regulates its binding and the level of monoubiquitinated H2A at K119 (ubH2AK119) at the *FOXO1* promoter. Thus, it increases *FOXO1* transcription and suppresses the growth of breast cancer [161].

### 2.5. Histone Sumoylation and Desumoylation

Small ubiquitin-like modifiers (SUMOs) have a similar three-dimensional structure with ubiquitin but differ by their flexible N-terminus, which also contains the site for SUMO chain formation. The conjugation of any SUMO member to a substrate is termed sumoylation. Similarly to ubiquitination, sumoylation is processed in a three-step enzymatic cascade that involves a dimeric E1 activating enzyme (SAE1 and SAE2), an E2 conjugating enzyme (Ubc9) and several SUMO E3 enzymes [220]. The sumoylation of histones is a highly dynamic process. A desumoylation is catalyzed by specific cysteine proteases, commonly called SUMO-specific isopeptidases or SUMO proteases. These enzymes cleave the isopeptide bond between the SUMO moiety and substrates. SUMO proteases belong to three distinct families, namely the Ulp/SENP, the Desi and the USPL1 family [230]. Through dynamic alterations of a substrate’s biochemical properties, sumoylation and its reverse reaction, desumoylation, can elicit rapid and reversible biological changes [231].

Sumoylation occurs predominantly in the nucleus and is involved in many processes, including signal transduction, transcription, protein subcellular localization and aggregation [232]. A number of studies have demonstrated the role of their deregulation in the development and progression of diseases [220].

The genes, coding proteins involved in sumoylation, also may be a target of ncRNA-mediated regulation. miR-200c was found to inhibit the expression of the SUMO-conjugating enzyme Ubc9 and Krϋppel-like transcription factor 4 (KLF4). Using a microRNA array screen, Zheng et al. determined that miR-200c expression is downregulated in vascular smooth muscle cells. Further experiments in cultured cells and animal models revealed that the miR-200c-sumoylated KLF4 feedback loop acts as a switch in transcriptional programs that control proliferation of vascular smooth muscle cells [233].

### 2.6. Histone Biotinylation and Debiotinylation

Biotinylation is one of the most recently described histone modifications. It represents covalent binding of the vitamin biotin to lysine residues in histones H2A, H3 and H4 and is mediated by biotinidase and holocarboxylase synthetase [234]. The determined sites of biotinylation are lysines K9, K13 and K129 in histone H2A, K4, K9 and K18 in histone H3 and K8 and K12 in histone H4 [234]. The acetylation of lysines and phosphorylation of serines were found to decrease biotinylation of adjacent lysine residues, whereas the dimethylation of arginine residues enhances the biotinylation of adjacent lysine residues [235]. The mechanisms of histone debiotinylation are largely unknown. It was suggested that biotinidase may catalyze both processes of histone biotinylation and debiotinylation [236]. Biotinylation of histones was confirmed to play a role in cell proliferation, gene silencing and cellular response to DNA damage. Biotinylated histones were detected in tumor cells in some types of cancer [237,238].

The biotinylation of histone H4K12 (H4K12bio) is associated with repression of a number of genes [239]. It is localized in transcriptionally repressed chromatin loci [240], and a low abundance of biotinylation marks is associated with genome instability in humans [239]

Bao et al. determined two miRNS, miR-539 and miR-153, involved in biotinylation regulation. Both of them can target holocarboxylase synthetase, which plays an essential role in catalyzing the biotinylation of histones in binding sites in the 3′ UTR of its mRNA. The effect of miR-153 suppression on the abundance of H4K12bio was confirmed in HEK-293 human kidney cells [241].

## 3. Histone Modifications and ncRNAs: From Theory to Praxis

Epigenetic mechanisms play an important role in virtually all pathological conditions, including neurological and cardiac disorders, autoimmune diseases and cancers. Besides, most of them appear already in the early stages of the pathogenic process and vary in different stages. Epigenetic regulators provide heritable phenotypic changes without alterations in DNA sequence; therefore, their aberrations are more flexible and reversible by pharmaceutical methods. All of that makes epigenetic regulators prominent diagnostic, prognostic and therapeutic markers. The choice of effective markers is important for clinical outcome, as they determine the possibility of early diagnosis and search for the relevant therapy. Some biomarkers may also be potential therapeutic targets [40].

Both ncRNAs and histone modifications have some advantages as biomarkers. The expression of ncRNAs is more tissue specific than expression of protein-coding genes. Being involved in pathogenesis of many diseases, they are often significantly deregulated [22]. Besides, some of them are differentially expressed in response to drug treatment and therefore can serve as predictive factors for the clinical response to therapies, thus opening novel opportunities for personalized medicine [242]. One of the most important characteristics of ncRNAs as a prominent biomarker is their stability. MiRNAs are stable in high-temperature, long-term storage and strong acidic or basic conditions and therefore could be detected in a wide range of samples, including formalin-fixed paraffin-embedded samples; they occur in body fluids in amounts significant for detection, so they could be used for noninvasive diagnostics [2]. CircRNAs are highly stable in the blood due to their circularized and covalently closed structure and ability to travel in the peripheral blood not only as cell-free RNAs but also within exosomes [243]. LncRNAs are frequently contained in exosomes or apoptotic bodies (P-bodies) that protect them even from fluids with high concentrations of ribonucleases. Besides, they could remain detectable in plasma even after several freeze–thaw cycles and prolonged incubation at elevated temperatures. However, because of their low expression levels, lncRNAs are difficult to use for diagnostic purposes in clinical practice [22]. There are a number of investigations devoted to the aberrant expression of ncRNAs in different diseases and associated with clinical characteristics of patients, which make them potential diagnostic and prognostic biomarkers. For example, low expression of miR-125a-3p correlates with tumor size, metastasis, invasion and poor prognosis in gastric cancer [244], and circVIM is upregulated in acute myeloid leukemia patients and associated with poor clinical outcome [245], whereas oncogenic lncRNA HOTAIR is upregulated in many tumors and promotes their progression [246].

Aberrant patterns of histone marks also play an important role in cancer pathogenesis. Thus, alterations in chromatin structure and histone marks, such as increased H3K4me2 and H3K18ac, could be associated with poor prognosis [247]. However, histone modifications are less prominent as biomarkers because of the technical limitations associated with their use as quantitative analytes and their lack of specificity for different diseases [193].

The application of epigenetic regulators as therapeutic target also seems to be a prospective direction. Epigenetic drugs (epi-drugs) are usually small-molecule inhibitors that are used for cancer treatment and achieve the therapeutic effects mainly by targeting and inhibiting epigenetic modifying enzymes and adjusting abnormal epigenetic changes. Epi-drugs can participate in the interruption of the cell cycle and the activation of tumor suppressor pathways, which in turn induce programmed cell death [248]. Besides, there are some non-pharmacological techniques of clinical management that are less obvious but could be effective for modulating ncRNA expression [40]. DNA methyltransferase inhibitors azacytidine (5-AZA) and decitabine (5-AZA-CdR) were the first epi-drugs approved by the United States Federal Drug Administration (FDA) in 2004 for the treatment of leukemia [249]. Currently, a number of epi-drugs that target different epigenetic mechanisms have been approved and even more are being developed and are undergoing clinical trials [162]. In addition to the DNMT inhibitors, among them are mostly EZH2 inhibitors and HDAC inhibitors. However, no LSD1 and PRMT inhibitors have been approved by the FDA [248].

HDAC inhibitors are divided into four groups based on their molecular mechanisms. There are valproic acids, carboxylic acids NaB and short-chain fatty acids, such as phenylbutyrate; hydroxamic acids, including trichostatin A (TSA) and suberoylanilide hydroxamic acid (SAHA, vorinostat); benzamides, such as entinostat (MS-275) and mocetinostat (MGCD-0103); and cyclic peptides, such as romidepsin (FK228). There are also an increasing number of HDAC inhibitors that have been developed, not included in any group [171]. Many HDAC inhibitors are non-specific and can be used to inhibit multiple isoforms of HDACs. HDAC inhibitors either cause global gene upregulation (potential tumor suppressors) and lead to the arrest of tumor cell growth, apoptosis and anti-angiogenesis, or they facilitate the binding of elongation factors to acetylated promoters and enhancers for efficient elongation, blocking gene elongation and inhibiting highly expressed genes (oncogenes) [250,251].

Most HDAC inhibitors are in clinical trials in phase I/II and have been approved by the FDA for different types of cancer [252]. Vorinostat (suberoylanilide hydroxamic acid, SAHA) is an oral HDAC inhibitor that has been approved by the FDA for the treatment of cutaneous T-cell lymphoma [248]. It binds to the zinc-containing pocket in the catalytic site of HDAC1, 2, 3 and 6, causing their reversible inhibition, stimulating stem cell differentiation to affect cell cycle and cell death [253]. Valproic acid (VPA) is a short-chain fatty acid oral antiepileptic agent that inhibits HDAC activity at low levels [254]. Romidepsin (depsipeptide) is a cyclic tetrapeptide that has proceeded through multicenter phase II trials as a therapy for refractory or relapsed AML [255]. Panobinostat was the first HDAC inhibitor approved for the treatment of multiple myeloma [256]. Another prominent therapy for cancer could be the combination treatment of HDAC and DNMT inhibitors for AML, glioma, breast cancer and CMML [257].

The EZH2 inhibitor tazemetostat selectively inhibits the activity of wild-type and mutant EZH2. It has been tested in a variety of cancers with functional deletion mutations of the SWI/SNF complex or abnormal activation of EZH2 resulting in histone hypermethylation, including sarcoma, non-Hodgkin’s lymphoma, medulloblastoma and many solid tumors [258,259], but it finally was approved by the FDA as the epi-drug for sarcoma in adults and children over the age of 16 with metastatic or locally advanced epithelioid sarcoma who are not suitable for radical surgery [248].

Epi-drugs could be used not only separately but also in combination with immunotherapy or traditional chemotherapeutic drugs [248]. The combination therapy has also been tested in a number of clinical trials and demonstrates prominent effect. For example, the combined application of vorinostat and the Wnt-β-linked protein blocker PKF118-310 in breast cancer facilitates the induction of cancer stem cells differentiation to inhibit the EMT process and reduce the number of breast cancer stem cells [260].

Epi-drugs are more commonly used in hematological tumors than in solid tumors. The limited efficacy of epi-drugs in solid tumors may be due to the high degree of cellular differentiation and intratumoral heterogeneity of solid tumors [261]. There are also a number of limitations in epi-drugs, which were not overcome yet. The most serious of them is the occurrence of adverse side effects. Therefore, it is necessary not only to find potential biomarkers for epi-drugs but also to exert their maximum efficacy at a minimum dose [248].

NcRNAs are also considered as the possible targets for therapy. The strategy of therapeutic miRNA alteration in cancer tightly depends on their function. Therefore, the level of oncogenic miRNAs that are overexpressed in tumors needs to be decreased, whereas tumor-suppressive miRNAs are downregulated in disease and require restoration of their expression [2]. The most common therapeutic approach for oncogenic miRNAs is the application of chemically modified anti-miRNA oligonucleotides (AMOS) that perfectly match to the specific mature miRNA, thereby preventing its functioning, or sponges that contain multiple binding sites to an miRNA of interest. Conversely, synthetic double-stranded miRNA mimics are complementary to the mRNA target of tumor-suppressor miRNAs and can restore their expression. Several such oligonucleotides have been already included in clinical trials as candidate therapeutics in different types of cancer [262].

SiRNAs are the exogenous counterparts to miRNAs and could be used to antagonize onco-miRs [263]. However, an important limitation for both siRNAs and miR-mimics is the difficulties associated with their delivery to tumor cells, as because of heavily hydrated phosphates on the surface of their liposome delivery vectors, they have difficulties with passage through hydrophobic cell membranes [264]. This limitation could be overcome by molecular modifications and the design of vehicles that aid specific delivery. Three therapeutic approaches are currently in development, namely lipid components, viruses and nanoparticles [265].

The strategy to decrease the lncRNA level in tumor cells is commonly their knockdown by siRNAs or small hairpin RNA (shRNA) via RNA interference. Another option is to use antisense oligonucleotides (ASOs). Upon interacting with target lncRNA, they form an RNA/DNA heteroduplex, which is further cleaved by endogenous RNaseH1 [2].

Among the limitations of application of ncRNAs in practice are a variability of miRNA expression in different conditions, which can result in biases even when a panel of several transcripts is used, as well as significant regional and rational differences of lncRNA profiling. The ability of one ncRNA to target multiple transcripts and proteins may lead to numerous side effects [2]. However, some ncRNAs are included in clinical trials for the treatment of various diseases, including various types of cancer, amyotrophic lateral sclerosis and polycystic kidney disease. The ncRNA entered into the clinical trial was a modified anti-sense oligonucleotide against miR-122 for the treatment of hepatitis C virus. As the drug Miravirsen (SPC3649), it is currently undergoing phase II clinical trials in several countries, including the US, Slovakia, the Netherlands and Germany [266]. The siRNA drug Patisiran was recently approved by the FDA to treat a polyneuropathy caused by hereditary transthyretin-mediated amyloidosis. It works by binding and degrading the transthyretin messenger RNA transcript [267].

Therefore, both histone modification and ncRNA demonstrate promising effects as biomarkers and targets for therapy. Even in clinical practice, their interactions could play an important role, as histone modification could be changed by targeting ncRNA, involved in its regulation, whereas alteration of histone marks pattern could modulate the expression of ncRNAs.

## 4. Conclusions

Epigenetics refers to the study of mechanisms that change gene expression without changing the primary DNA sequence. Over the last three decades, an increasing number of investigations have allowed for concluding their important role in virtually all processes in cells. Nevertheless, a comprehensive picture of epigenetic patterns both in normal cells and in different diseases is still emerging. An understanding of the mechanisms underlying their functioning is even more challenging and urgent. Some of these mechanisms were already described. Thus, DNA methylation is a relatively well-investigated process to date, and the protein complexes involved in histone acetylation are well studied. Even ncRNAs that were only recently included in the system of the epigenetic regulation are no longer considered to be the “dark matter” of the genome. However, it has already become clear that epigenetic regulators are not working alone, but rather they are tightly connected and form a comprehensive network of regulatory pathways and feedback loops. That makes the gap in our knowledge even wider and the challenge even more fascinating.

The deregulation of epigenetic mechanisms was observed in early stages of numerous diseases. Besides, in contrast to genetic changes, which are difficult to reverse, epigenetic mechanisms are heritable and reversible. Their early appearance and flexibility make them a prominent tool for diagnostics, prognosis and therapy. The improvements in understanding of epigenome organization and mechanisms allowed the development of epi-drugs and methods for modulation of ncRNA expression. Several of such epi-drugs and biomarkers have been approved by the FDA and are already used in clinical practice. However, the limitations of the delivery systems and adverse side effects do not allow their wide clinical application. Therefore, despite the substantial progress in the investigation of epigenetic regulation, further efforts are still required.

## Figures and Tables

**Table 1:** Types of histone modifications and their effect on transcription.

Modification	Group	Effect on Transcription	Sites of Modification
1. Modifications by small chemical groups			
Methylation	Methyl group	Activation	H3 (K4, K36, K79)
		Repression	H3 (K9, K27), H4K20
Acetylation	Acetyl group	Activation	H3 (K4, K9, K14, K18, K27, K56); H4 (K5, K8, K12, K16); H2A/H2B (K6, K7, K16, K17)
Phosphorylation	Phosphate	Activation	H3 (S10)
2. Modifications by complex molecules			
Ubiquitination	Ubiquitin	Activation	H2B (K120)
		Repression	H2A (K119)
Sumoylation	SUMO	Repression	H4 (K5, K8, K12, K16), H2A (K126), H2B (K6, K7, K16, K17)
Biotinylation	Biotin	Repression	H2A (K9, K13, K129), H3 (K4, K9, K18), H4 (K8, K12)

**Table 2:** NcRNAs, regulating histone modifications and their role in pathogenesis.

NcRNA	Target Modification	Epigenetic Regulatory Mechanisms	Role in Pathogenesis	Ref.
	Methylation/Demethylation			
ANRIL	H3K27me3	Directly interacts with PRC2 (EZH2 and SUZ12)	Promotes cholangiocarcinoma progression	[47]
CANT1	H3K4me3	Prevents hSET1 from binding to the PI3Kγ promoter	Suppresses retinoblastoma progression	[48]
Chaer	H3K27me3	Interacts with PRC2	Promotes cell proliferation and induces apoptosis in atherosclerosis	[49]
circ_0019435	H3K27me3	Directly binds EZH2 and silences the expression of DKK1 and PTEN	Promotes proliferation, invasion and EMT in cervical cancer	[50]
circAGFG1	H3K27me3	Recruits EZH2 to the promoter of p53 and inhibits its expression	Regulates proliferation and apoptosis in cervical cancer	[51]
DLEU1	H3K4me3	Activates JAK-STAT signaling pathway	Promotes oral squamous cell carcinoma progression	[52]
FEZF1-AS1	H3K4me, H3K4me2	Specifically binds LSD1, regulating the expression of CDKN1A (P21)	Involved in pathogenesis of colorectal carcinoma and glioma; promotes gastric cancer proliferation	[53,54,55]
Firre	H3K27me3	Interacts with SUZ12	Maintains X chromosome inactivation	[56]
GAS8-AS1	H3K4me3	Recruits the MLL1/WDR5 complex and maintains the GAS8 promoter in an open chromatin state	Inhibited malignant transformation of hepatocytes	[57]
H19	H3K27me3, H3K4me3	Facilitates the PRC2 complex in regulating methylation changes at H3K27me3/H3K4me3 histone sites of genes; represses VASH1 through EZH2-dependent H3K27 trimethylation	Regulates tumor plasticity in neuroendocrine prostate cancer; important regulator in human amniotic mesenchymal stem cells for promoting angiogenesis	[58,59]
HOTAIR	H3K27me3	Recruits PRC2 and mediates H3K27me3 in different genes	Promotes proliferation, invasion, migration in tumors; promotes EMT in gastric cancer; promotes the self-renewal of leukemia stem cells; promotes metastasis of renal cell carcinoma	[60,61,62,63]
HOXA-AS3	H3K27me3	Facilitates EZH2-mediated H3K27me3, target RUNX2	Inhibits osteogenic differentiation of mesenchymal stromal cells, regulates degenerative bone diseases	[45]
IL6-AS1	H3K4me	Promotes IL-6 transcription by recruiting early B-cell factor 1 to the IL-6 promoter	Associated with airway inflammation	[64]
LAMP5-AS1	H3K79me2, H3K79me3	Directly binds DOT1L, promoting the global patterns of H3K79me2/me3 in cells	Regulates the self-renewal program and differentiation block in MLL leukemia	[65]
let-7i	H3K9me2	Targets KDM3A, thus removing the DCLK1 H3K9me2, and suppresses the FXYD3	Lung cancer progression	[66]
LINC01419	H3K27me3	Binds to EZH2, leading to histone methylation of the RECK promoter	Hepatocellular carcinoma growth and metastasis	[67]
LINP1	H3K27me3	Recruits EZH2 to the promoter regions of tumor suppressors KLF2 and PRSS8	Regulates proliferation and apoptosis in cervical cancer	[68]
lnc-ATB	H3K27me3	Directly interacts with EZH2	Regulates proliferation, invasion and migration; promotes ovarian cancer	[69]
lnc-OB1	H3K27me3	Upregulates OSX via the inhibition of H3K27me3 in the OSX promoter; interacts with SUZ12	Promotes osteogenic differentiation in human osteoblasts, might be a drug target for the treatment of osteoporosis	[70]
lncRNA CIR	H3K27me3	Binds to EZH2 and repressed ATOH8 expression via EZH2-mediated H3K27me3, which promotes the methylation of ATOH8	Inhibits chondrogenic differentiation	[71]
lncRNA ROR	H3K4me3	Recruits histone transmethylase MLL1 to upregulate TIMP3 expression	Promotes breast cancer progression	[72]
lncRNA-p21	H3K27me3	Switches the EZH2 function from histone methyltransferase to non-histone methyltransferase, consequently methylating the STAT3	Promotes the neuroendocrine differentiation in antiandrogen enzalutamide-induced prostate cancer	[73]
MALAT1	H3K27me3	Recruits EZH2 to promoters of target genes, facilitating H3K27me3	Potentiates growth and inhibits senescence in gallbladder cancer cells; indicates poor prognosis; releases epigenetic silencing of HIV-1 replication; regulator of inflammation in diabetic retinopathy	[74,75,76]
MEG3	H3K27me3	Recruits EZH2; increases H3K27me3 via P53; inhibits the expression of TERT by increasing the H3K27me3 in TERT promoter regions and inhibition of the activity of telomerase by reducing the binding of TERT to TERC; increases the expression of TERRA	Reduces the activity of telomerase and blocks telomere	[77,78,79]
miR-101	H4K27me3, H3K9me3, H4K20me3	Directly targets EZH2	Regulates autophagy, proliferation and apoptosis in laryngeal squamous cell carcinoma; affects endothelial function and angiogenesis in diabetes mellitus	[80,81]
miR-124	H3K27me3	Directly targets EZH2	Promotes tumor growth and is associated with poor prognosis in esophageal squamous cell carcinoma; involved in thyroid carcinoma pathogenesis, reduced in sorafenib insensitive patients	[82,83]
miR-125b	H3K9me3	Targets Suv39h1	Sustains inflammation in diabetes	[84]
miR-137	H3K4me2, H3K4me3	Directly targets EZH2, JARID1B, KDM5B	Correlated with the poor prognosis and short-term survival of patients with cervical cancer; inhibits cell proliferation in acute lymphoblastic leukemia; involved in PTEN-Null prostate cancer	[85,86]
miR-152-3p	H3K9me3	Targets SETDB1	Positively regulates the production of H3K9me3	[87]
miR-155	H3K27me3	Crosstalk between miR-155-PRC2 complex-JARID2 and PHF19	Involved in endometriosis	[88]
miR-193a	H3K4me, H3K4me2 and H3K4me3	Directly targets the MLL1	Regulates cell growth, migration and apoptosis	[89]
miR-195–5p	H3K27me3	Inhibits EZH2	Promotes tubular injury in diabetic nephropathy	[90]
miR-199b-3p	H3K27me3	Increases E-cadherin expression and prevents EMT through repressing KDM6A expression	Protects renal tubules from diabetic-induced injury	[91]
miR-214	H3K27me3	Directly targets EZH2	Low expression correlates with advanced stage and poor overall survival of patients with cervical cancer	[92]
miR-216b	H3K9me2, H3K9me3	Directly targets JMJD2C, downregulation of the JMJD2C/HIF1α/HES1 signaling axis	Positively correlated with patient survival in osteosarcoma, enhanced cisplatin-induced apoptosis	[93]
miR24-2	H4R3me2, H4R3me3	Targets PRMT7	Associated with human tumorigenesis	[94]
miR-29	H4K20me3	Directly suppress Suv4-20h	Contributes to cellular senescence and cardiac aging	[95]
miR-29a	H4K20me3	Downregulates histone H4K20 trimethylation through directly targeting SUV420H2	Involved in breast cancer cells epithelial–mesenchymal transition, migration and invasion	[96]
miR-506-3p	H3K27me3	Directly targets EZH2	Involved in thyroid carcinoma pathogenesis, reduced in sorafenib insensitive patients	[83]
miR-7	H4K20me1	Negatively regulates SET8	Suppresses the invasive potential of breast cancer cells and sensitizes cells to DNA damage	[97]
miR-93-5p	H3K9me3	Directly suppresses Bcl-w	Inhibits premature cellular senescence	[98]
NEAT1	H3K9me2	Transcriptional repression of the c-Fos by H3K9me2 at promoter; binds to EZH2	Mediates age-related memory impairment; contributes to glioblastoma progression	[99,100]
PAN RNA	H3K27me3	Interacts with UTX and JMJD3, removes the repressive marks on the chromatin	Involved in Kaposi’s sarcoma-associated herpesvirus-mediated malignancies	[101]
PART1	H3K27me3	Epigenetic silencing of PDGFB via the PLZF-mediated recruitment of EZH2	Restrains aggressive gastric cancer; low expression is associated with postoperative metastasis and short overall survival	[102]
PAS1	H3K9	Recruits SUV39H1 to methylate H3K9 of PH20	Inhibits breast cancer growth and metastasis	[103]
PHACTR2-AS1	H3K9me2, H3K9me3	Directly binds ribosome DNA genes and recruits SUV39H1	Promotes growth and metastasis in breast cancer	[104]
PVT1	H3K27me3	Forms a complex with EZH2, directly binding the promoter region of miR-195	Related to large tumor size, advanced stage and poor prognosis in cervical cancer; regulates the chemoresistance	[105]
SNHG1	H3K27me3	Interacts with EZH2 and acts as a sponge for miR-154-5p and miR-143-3p	Oncogenic functions in colorectal and bladder cancer	[106,107]
SNHG22	H3K27me3	Binds EZH2 and regulates miR-200c-3p/Notch1 axis	Promotes proliferation and invasion, poor prognosis in gastric cancer	[108]
SNHG7	H3K27me3	Recruits EZH2 to promoter of the inhibitor of the Wnt/β-catenin signaling DKK	Promotes cervical cancer	[109]
SNHG8	H3K27me3	Directly interacts with EZH2, inhibiting the expression of RECK at the transcriptional level	Promotes cervical cancer	[110]
SSTR5-AS1	H3K4me3	Interacts with MLL3 and increases the enrichment of MLL3 and H3K4me3 at the promoter region of SSTR5	Inhibits laryngeal carcinoma cells proliferation, migration and invasion	[111]
ST3Gal6-AS1	H3K4me3	Binds MLL1 and recruits it to the ST3Gal6 promoter region	Mediates colorectal cancer progression	[112]
TGFB2-AS1	H3K27me3	Binds to the EED adaptor of the PRC2	Regulates TGF-β signaling	[113]
UCA1	H3K27me3	Interacts with the EZH2 and suppresses p21 expression	Promotes breast cancer progression; expression is higher in tamoxifen-resistant breast cancer cells	[114]
XIST	H3K27me3	Interacts with EZH2 and downregulates DKK1	Facilitates cell growth, migration and invasion in neuroblastoma	[115]
**Acetylation/deacetylation**				
ANCR	H3ac, H4ac	Inhibits HNRNPA1 degradation and sponging miR-140-3p	Promotes hepatocellular carcinoma metastasis	[116]
ANRIL	H3K9ac	Directly recruits PRC1 and PRC2	Associated with the development of cardiovascular diseases	[117,118]
CircITCH	H3K9ac	Sponge of miR-330-5p that upregulates SIRT6, Survivin and SERCA2a	Regulates DNA damage, mitochondrial oxidative stress, cell death, calcium handling defects and contractile dysfunction in DOX-treated human-induced pluripotent stem-cell-derived cardiomyocytes	[119]
circMRPS35	H4K5ac	Enriches H4K5 acetylation at the regions of FOXO1 and FOXO3a promoters by recruiting the histone acetyltransferase KAT7	Inhibits invasion and proliferation of gastric cancer cells, related to the clinicopathological features and better prognosis of patients	[120]
circMYO10	H4K16Ac	Regulates miR-370-3p/RUVBL1 axis	Promotes osteosarcoma progression	[121]
DLEU1	H3K27ac	Upregulates interferon-stimulated genes (IFIT1, IFI6 and OAS1ISGs) through activation of JAK-STAT signaling	Promotes oral squamous cell carcinoma progression	[52]
DSCAM-AS1	H3K27ac	Promotes DCTPP1 gene transcription by affecting H3K27 acetylation and enhanced DCTPP1 mRNA stability by binding to the 3′ untranslated region	Suppresses the growth and invasion of ER-positive breast cancer cells	[122]
EPB41L4A-AS1	H3K27ac, H3K14ac	Binds to GCN5 and activates transcription of TXNIP	Regulates glucose uptake	[123]
GAS5	H3K9ac	GAS5 upregulates SIRT1 via inhibition of miR-221	Inhibits cell proliferation and fibrosis in diabetic nephropathy	[124]
H19	H3ac, H4ac	Sponge of miR-19b, which targets SIRT1	Involved in diabetic retinopathy; promotes neuroinflammation in ischemic stroke	[59,125]
HOTAIR	H3K27ac	Downregulates miR-761 and increases HDAC1 expression; HOTAIR/miR-34A axis mediates SIRT1 expression	Promotes progression and angiogenesis of endometriosis; oncogenic role in tumors	[61,126]
HOTAIRM1	H3K27ac	Positively modulates the activity of c-Jun, which recruits the acetyltransferase p300 to RUNX2 promoter and activates the gene	Promotes osteogenesis	[127]
IL6-AS1	H3K4me, H3K27ac	Promotes IL-6 transcription by recruiting early B-cell factor 1 to the IL-6 promoter	Associated with airway inflammation	[64]
KTN1-AS1	H3K27ac	Recruits EP300 to the KTN1 promoter region	Promotes bladder cancer tumorigenesis	[128]
LINC-00162	H3ac	Promotes HDAC9 via inhibition of miR-383	Participates in the pathogenesis of diabetic nephropathy	[129]
LINC00930	H3K9ac	Scaffold to recruit the RBBP5 and GCN5 complex to the PFKFB3 promoter	Associated with tumorigenesis, lymphatic invasion, metastasis and poor prognosis in nasopharyngeal carcinoma	[130]
lincRNA-p21	H3ac, H4ac	Inhibits acetylation of H3 and H4 at the Thy-1 promoter and Thy-1	Could lead to pulmonary fibrosis in acute respiratory distress syndrome	[131]
Lnc34a	H3ac, H4ac	Suppresses miR-34a through recruiting HDAC1 to promote histones deacetylation	Significantly overexpressed in hepatocellular cancer and associated with bone metastasis	[132]
LncPRESS1	H3K56ac, H3K9ac	Interacts with SIRT6	Maintains embryonic stem cells’ pluripotency	[133]
MALAT1	H3ac	Reduces the H3 histone acetylation of the PDX-1 promoter and subsequently inhibits the expression of PDX-1, thus suppressing insulin secretion	Induces the dysfunction of β cells in type 1 diabetes	[134]
miR-149-5p	H4K16ac	Regulates KAT8 and H4K16ac expression	Involved in an Alzheimer’s disease pathogenesis	[64]
miR-15a-5p	H4ac	Suppresses acetyl-CoA activity and decreases histone H4 acetylation by inhibiting ACSS2 expression	Inhibits metastasis and lipid metabolism	[135]
miR-193b-3p	H3ac	Directly targets HDAC3	Regulates chondrogenesis and chondrocyte metabolism	[136]
miR-196-b	H3ac, H4ac	Targets ING5	Closely linked to the tumor size of neuroblastomas	[137]
miR-205	H3K9ac	Directly targets HDAC2	Regulates extracellular matrix production in tubular epithelial cells in individuals with diabetic kidney disease	[138]
miR-22	H3K56ac	Directly binds to SIRT1	Regulator of stress-induced heart damage	[139]
miR24-2	H4K16ac	Targets PRMT7; inhibits HDAC3 through miR-675	Associated with human tumorigenesis	[94]
miR-29a	H3K27ac	Binds to RANKL	Represses osteoclast formation and protects against osteoporosis	[96]
miR-29b	H3K14ac	Targets HDAC4	Mediates podocyte dysfunction and renal fibrosis in diabetic nephropathy	[140]
miR-376a	H3K18ac	Targets HDAC9	Contributes to the development of hepatocellular carcinoma	[141]
miR-449	H3K4ac, H3K9ac	Interacts with HDAC1	Improves cardiac function	[142]
miR-455-3p	H3ac	Directly targets HDAC2/8	Modulates cartilage development and degeneration	[143]
miR-494	H3K9ac	Inhibits HDAC3 in neurons	Involved in ischemic stroke pathogenesis	[144]
miR-520b	H3K9ac	Directly targets HDAC4	Accelerates lung cancer cell proliferation	[145]
miR-543	H3ac, H4ac	Targets ING5	Closely linked to the tumor size of neuroblastomas	[137]
miR-766-5p	H3K27ac	Directly targets CBP and BRD4, reducing levels of H3K27ac at MYC super-enhancers	Protumorigenic	[146]
miR-92a-3p	H3ac	Directly targets HDAC2	Regulates cartilage development and homeostasis	[147]
miR-N5	H3K56ac	Targets CREBBP, mediating H3K56 acetylation at the promoter of EGFR, β-catenin and CDH1	Inhibits metastasis of prostate cancer	[148]
NEAT1	H3K27ac	Sponges miR-524-5p, miR-221-3p	Promotes the proliferation and invasion of laryngeal cancer cells; associated with neurodegenerative disorders	[100,149,150,151,152]
SATB2-AS1	H3K27ac, H3K9ac	Acts as a scaffold to recruit p300	Suppresses colorectal carcinoma progression	[153]
SNHG14	H3K27ac	Upregulates PABPC1 through H3K27 acetylation and modulates PTEN signaling; activates Nrf2 signaling pathway	Promotes hepatocellular carcinoma progression; induces trastuzumab resistance of breast cancer	[154,155]
TINCR	H3K27ac	Bind ACLY, starting the TINCR-ACLY-PADI1-MAPK-MMP2/9 axis	Promotes nasopharyngeal carcinoma progression and chemoresistance	[156]
TUG1	H3K9ac	Sponges miR-132-3p, activating HDAC3	Mediates ischemic myocardial injury	[157]
**Phosphorylation/dephosphorylation**				
miR-93	HS10	Targets a histone kinase Msk2	Diabetic nephropathy progression	[158]
**Ubiqiutinylation/deubiquitinylation**				
let-7b	H2BK120ub1	Binds directly and inhibits USP42, USP44, ATXN7L3	Tumor-suppressive effects	[159]
let-7c	H2BK120ub1	Binds directly and inhibits USP42, USP44, ATXN7L3	Tumor-suppressive effects	[159]
linc-RA1	H2BK120ub1	Binds with H2B and inhibits the interaction between H2Bub1 and USP44	Associated with advanced clinical stage of glioma, promotes glioma radioresistance	[160]
lncFOXO1	H2AK119ub1	Regulates level ubH2AK119 at FOXO1 promoter	Suppresses growth of human breast cancer cells	[161]

## Data Availability

Not applicable.
